# One-Stage Reconstruction of Scalp after Full-Thickness Oncologic Defects Using a Dermal Regeneration Template (Integra)

**DOI:** 10.1155/2015/698385

**Published:** 2015-11-16

**Authors:** Barbara De Angelis, Pietro Gentile, Eleonora Tati, Davide J. Bottini, Ilaria Bocchini, Fabrizio Orlandi, Giampiero Pepe, Chiara Di Segni, Giulio Cervelli, Valerio Cervelli

**Affiliations:** ^1^Department of Plastic and Reconstructive Surgery, University of Rome “Tor Vergata”, 00133 Rome, Italy; ^2^Catholic University “Our Lady of Good Counsel”, Tirana, Albania

## Abstract

The use of Dermal Regeneration Template (DRT) can be a valid alternative for scalp reconstruction, especially in elderly patients where a rapid procedure with an acceptable aesthetic and reliable functional outcome is required. We reviewed the surgical outcome of 20 patients, 14 (70%) males and 6 (30%) females, who underwent application of DRT for scalp reconstruction for small defects (group A: mean defect size of 12.51 cm^2^) and for large defects (group B: mean defect size of 28.7 cm^2^) after wide excision of scalp neoplasm (basal cell carcinoma and squamous cell carcinoma). In group A, the excisions were performed to the galeal layer avoiding pericranium, and in group B the excisions were performed including pericranium layer with subsequent coverage of the exposed bone with local pericranial flap. In both the groups (A and B) after the excision of the tumor, the wound bed was covered with Dermal Regeneration Template. In 3 weeks we observed the complete healing of the wound bed by secondary intention with acceptable cosmetic results and stable scars. Scalp reconstruction using a DRT is a valid coverage technique for minor and major scalp defects and it can be conducted with good results in elderly patients with multiple comorbidities.

## 1. Introduction

Reconstruction of the scalp after tumor excision may include any of the following techniques: secondary granulation [1]; side-to-side closure [2]; the use of an advancement flap [3], rotation flap [4], or transposition flap [5]; tissue expansion; locoregional [6] or free flaps [7–9]; or the use of a split-thickness skin graft (STSG) or full-thickness skin graft (FTSG) [10]. Choosing one technique depends on anatomic and patient-related factors. Anatomic factors include the laxity of local tissue surrounding the defect and the depth of the wound bed. Patient-related factors include physical and mental conditions, comorbidities, patient's expectations, and ability to take care of the reconstructed defect.

Granulation of the wound bed is dedicated to patients that have the desire of minimal amount of surgery, do not have the ability of an adequate postoperative care, and have comorbidities factors that interfere with the wound healing. This healing technique is only dedicated to superficial defects especially without bone exposure.

Good candidates for side-to-side closure are patients with very small defect that can be closed without significant tension and can be easily closed primarily [2].

Local flaps [11] (rotation, advancement, and transposition flaps) can be useful for reconstruction of small defect size; to create flaps, redundant tissue surrounding the wound is required; the scalp usually does not have significant redundant tissue to harvest local flaps. Sometimes tissue expansion can be useful for reconstruction using local flap but the discomfort for the patient is very high and this technique cannot be dedicated to elderly patients [12, 13]. In general for large defects of the scalp random pattern flaps [14, 15] have limited applications and locoregional or free flaps are preferred. Anyway these last two types of flaps have high risk of complications especially in old patients considering factors related to the surgery technique and to the postoperative care; sometimes for patients with multiple comorbidities and large defect size after tumor excision the best choice is skin grafting [10]. Anyway, skin grafts can be prone to contraction and they leave a contour deficit and are less reliable and robust when used on large areas of exposed bone because they can easily necrotize and be infected [10].

We can assume that reconstruction of large scalp defects especially in elderly patients with multiple comorbidities presents a challenge for the surgeon. Actually for these patients a valid solution can be the use of Dermal Regeneration Template to cover defects of the scalp [16].

The Integra Dermal Regeneration Template (DRT) was introduced into clinical practice in Europe in 1996 by Yannas and Burke's component analysis in 1970 [17–19].

Since its development DRT has been mainly used in burns wounds and for the treatment of chronic wounds with great success [20]; from 2005 DRT has been used to cover complex scalp defects following excision of malignant tumors [21].

Dermal Regeneration Template is an artificial dermis manufactured as a synthetic bilaminate (Integra Double Layer) composed of a bovine collagen lattice covalently linked to chondroitin-6-sulfate and covered with a synthetic polysiloxane polymer (silicone) epidermis [22].

Fibroblasts, macrophages, lymphocytes, and endothelial cells migrate from surrounding host tissue into the artificial dermis, vascularize it, and, finally, replace it.

The usual practice for coverage of scalp defect with Integra Double Layer is the excision of small tumors sparing pericranium, apposition of DRT subsequently after the removal of silicone positioning of thin skin grafting; instead, in large defects the excision of tumor is conducted to the pericranium, after burring the bone with drill, covering the bone with DRT, and subsequently positioning of the skin graft after the removal of the silicone layer [23–25].

In our paper, we studied 20 elderly patients with multimorbidities and with extended defects of the scalp area who had been treated with Integra between July 2013 and June 2014 at the Department of Plastic Surgery, Policlinico Casilino, University of Rome Tor Vergata.

We did not cover the DRT after the removal of silicone layer with skin graft but we evaluated the effective result of reepithelization by secondary intention.

We also evaluated a new technique for coverage of the exposed calvaria bone after wide tumor excision with local pericranial flap and its important role in the attachment of DRT to the bone.

## 2. Patients and Methods

Between July 2013 and June 2014, 20 patients (14 males and 6 females), mean age 80 years, affected by epithelial scalp cancer (4 with squamous cell carcinoma (SCC) and 16 with basal cell carcinoma (BCC)) were treated at Plastic and Reconstructive Surgery Division of Policlinico Casilino, Tor Vergata University.

We divided the patients into two groups with two different surgical treatments: group A and group B.

In group A, we treated 10 patients with mean defect size of 12.51 cm^2^  ±  4.4 (SD) and maximum defect size of 19.62 cm^2^; in group B we treated 10 patients with mean defect size of 28.7 cm^2^  ±  13.5 (SD) and maximum defect size of 65.94 cm^2^ (Tables 1 and 2).

In this study we chose elderly patients older than 70 years with multiple concomitant diseases such as arterial hypertension, coronary heart disease, and atrial fibrillation; under antiplatelet drugs and permanent anticoagulation therapy; and with chronic renal failure, diabetes mellitus type II, smoking habits, and chronic lymphocytic leukemia. We excluded from our study patients with nonepithelial skin cancer.

We also excluded patients under the age of 70 or in good conditions as for them other types of reconstruction are suggested such as local flaps or free tissue transfer.

The subjective and objective assessment of the scars was performed using the Manchester Scar Scale (MSS). The Manchester Scar Scale was introduced by Beausang et al. in 1998. The scale has five parameters for the evaluation of the scar: color, skin texture, contour, distortion, and texture giving a score of 1 to 4 for all the parameters except for skin texture that is represented by score 1 or 2 (matte or shiny) and a Visual Analogue Scale (VAS) that describes the overall cosmetic appearance of the scars from excellent to poor, giving a score from 0 to 10. Scores from the 2 evaluations (five parameters plus VAS) are added together to give an overall score for the scar; the score range is from a minimum value of 4 for the best clinical scar to 28 representing clinically worse scar, which are shown as follows [26–28].

*The Manchester Scar Scale (MSS) and a Visual Analogue Scale (VAS).* They are as follows.

 Excellent—Poor
  1. *Color (cf. to surrounding skin)*
    1. Perfect—1
    2. Slight mismatch—2
    3. Obvious mismatch—3
    4. Gross mismatch—4
  2. *Skin texture*
    1. Matte (1)/Shiny (2)
  3. *Contour*
    1. Flush with surrounding skin—1
    2. Slightly proud/indented—2
    3. Hypertrophic—3
    4. Keloid—4
  4. *Distortion*
    1. None—1
    2. Mild—2
    3. Moderate—3
    4. Severe—4
  5. *Texture*
    1. Normal—1
    2. Just palpable—2
    3. Firm—3
    4. Hard—4.

### 2.1. Surgical Procedure

#### 2.1.1. First Step

The procedures were carried out under local anesthesia for patients included in group A and under general anesthesia in group B.

A broad-spectrum intravenous antibiotic was given at induction and the administration was continued for the next 48 hours during hospitalization.

**Figure 1:**
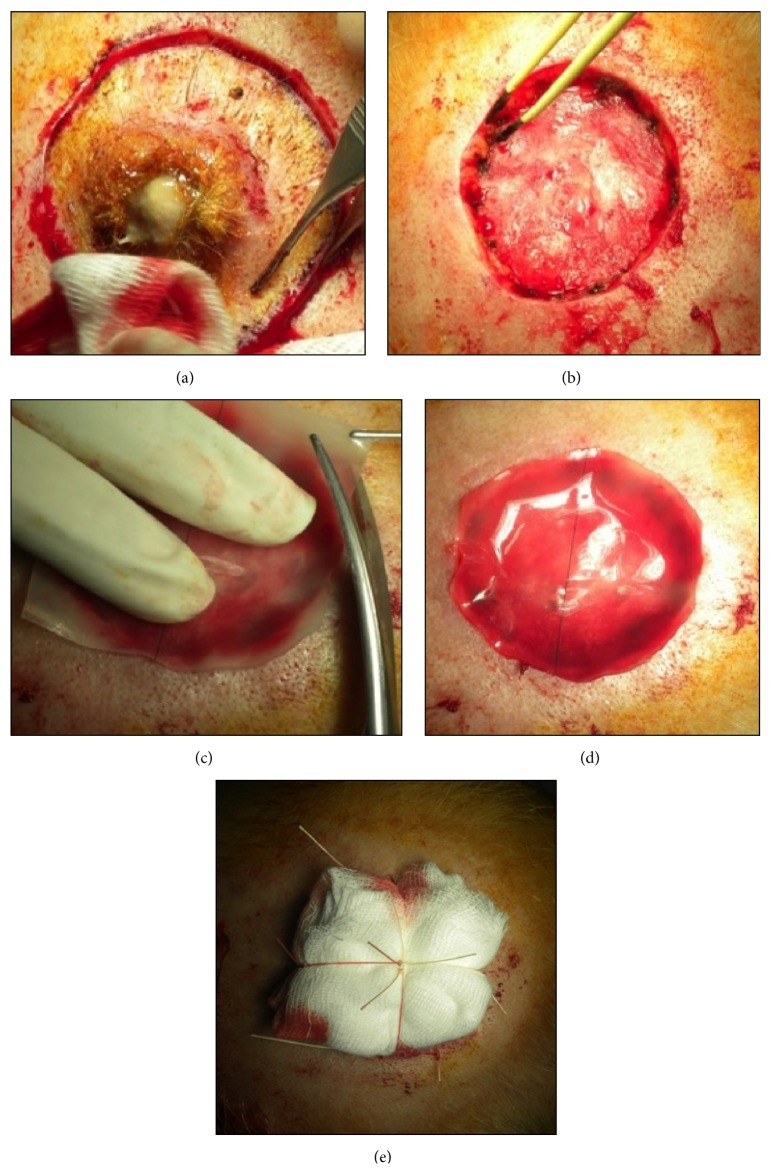
Patient of group A: (a) excision of the lesion including the galeal layer, avoiding the pericranium, (b) detail of loss of substance after the excision, (c) remodelling of a single piece of Integra Double Layer (DL) (5 cm × 5 cm), (d) Integra after remodelling which has then been shaped to size, and (e) moulage.

**Figure 2:**
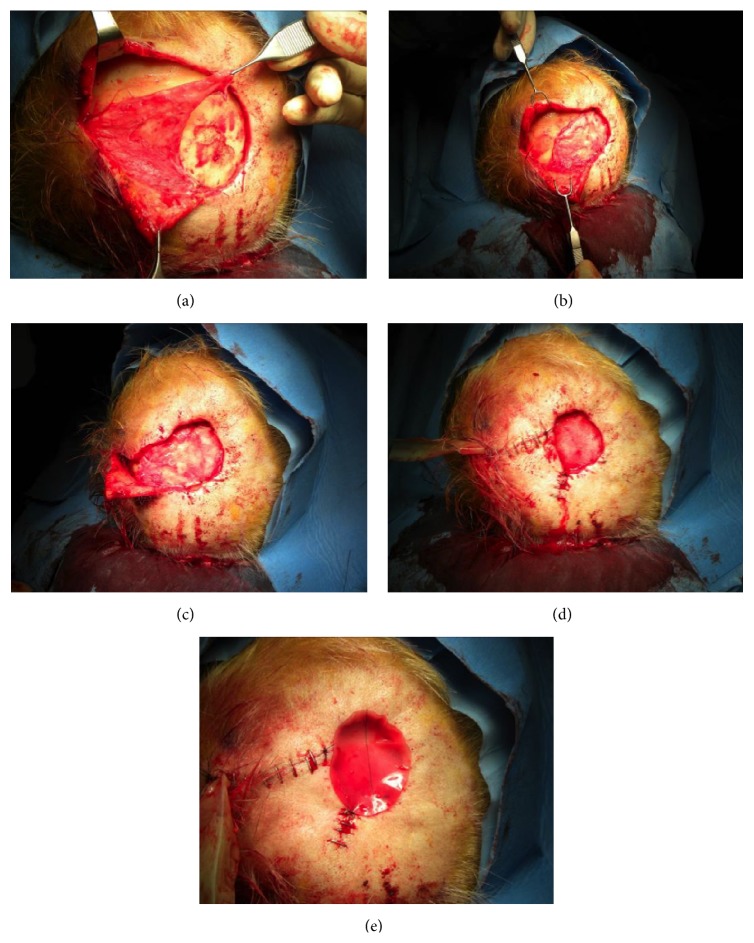
Patient of group B: (a) excision of the lesion including the pericranium, (b) harvesting of local pericranial flap, (c) phase of pericranial flap rotation, (d) local flap fixed, and (e) coverage with Integra Double Layer which has then been shaped to size.

For all patients the hospitalization was at least 48 hours regarding general conditions of each patient. In group A the excisions were performed to the galeal layer, avoiding the pericranium; in group B the excisions were performed including pericranium layer with exposure of the calvaria bone. After the excision the bed of the lesion was firstly checked for an accurate hemostasis; in group A the lesions were immediately covered with a single piece of Integra Double Layer (DL) (5 cm × 5 cm), which has then been shaped to size (Figure 1), and in group B the lesions were covered with a local rotational pericranial flap and with a single piece of Integra Double Layer (DL) (10 cm × 12.5 cm) which has then been shaped to size (Figure 2).

The removed tissue was marked for its orientation and sent for histology.

The Dermal Regeneration Template was then fenestrated with a scalpel blade 11 in order to reduce the accumulation of fluids. The sheets were applied to wound edges by 4-0 nonabsorbable sutures and secured with staples.

Pressure dressing and occlusive bending were applied above Integra. Neither VAC therapy nor antiseptic medications have been used.

24 hours postoperatively the patient has taken the semisitting position (45°) in order to reduce the risk of hematomas.

Early discharge from hospital was encouraged; after the 48 hours of intravenous antibiotic therapy an oral therapy for 5 days was established.

Special care has been paid to the control of the wound bed in the days immediately following surgery in order to find any early signs of infection or side effect of the therapy.

The patients were visited after 5 days, and the outer dressing was removed for the first time to find out any potential or developing complications.

#### 2.1.2. Second Step

**Figure 3:**
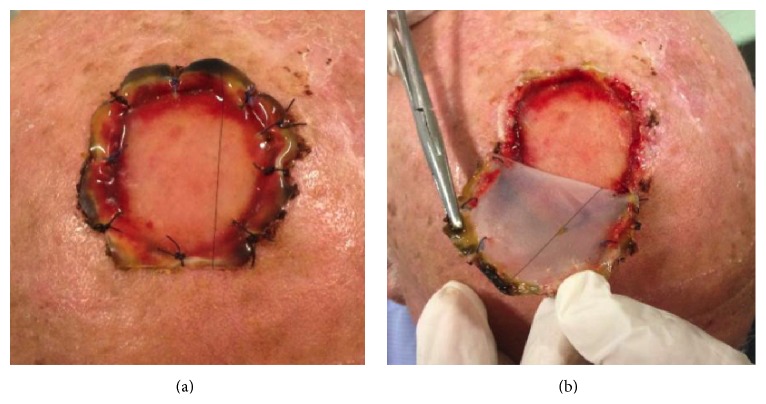
(a) Integra Double Layer after sixteen days and (b) removal of the silicone layer of Integra.

After 3 weeks the template switched its color from dark red to light pink or vanilla, sign of a correct development of new derma. The outer silicone layer was peeled off, and the area was completely cleaned (Figure 3).

Excess granulation tissue was removed.

We educated the patients to make by themselves daily medications with hyaluronic acid gauze dressings and we ambulatorily checked the patients once a week until complete wound healing. For all patients clinical follow-up lasted 3 months, but for patients with histological diagnosis of squamous cell carcinoma a further ultrasonography of nodal stations was performed at the time of diagnosis and after 6 months from the reconstructive surgery.

## 3. Results

**Figure 4:**
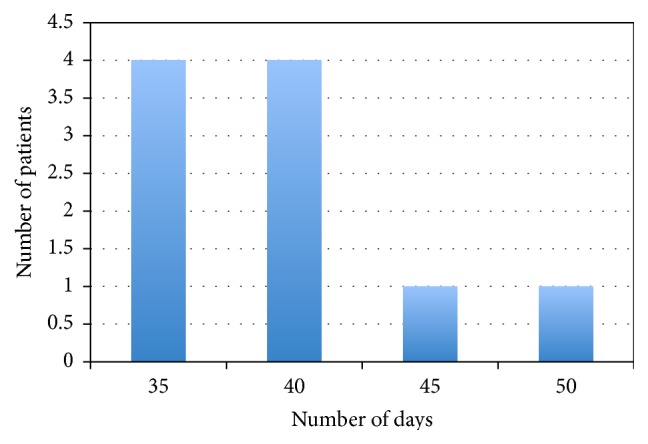
Patient of group A: reepithelization time.

**Figure 5:**
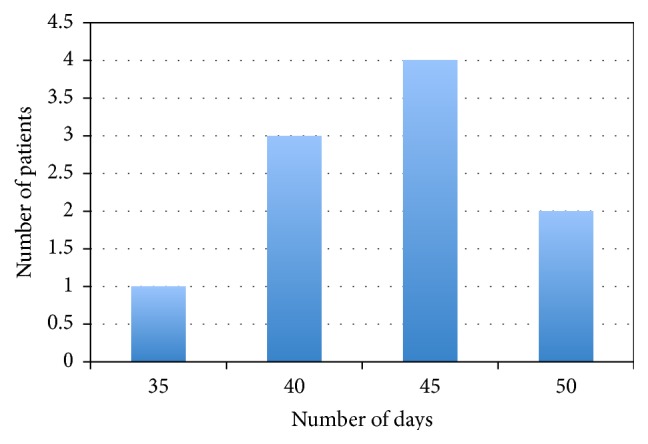
Patient of group B: reepithelization time.

In group A the complete reepithelization time was achieved after 35 days postoperatively in 4 cases, 40 days in 4 cases, 45 days in 1 case, and 50 days in 1 case (Figure 4); in group B the complete reepithelization time was achieved after 35 days in 1 case, 40 days in 3 cases, 45 days in 4 cases, and 50 days in 2 cases (Figure 5). The mean postoperative reepithelization time was 42.5 ± 6.4 (SD) days for both groups.

The results of the Manchester Scar Scales (MSS) are summarized in Tables 3 and 4.

**Figure 6:**
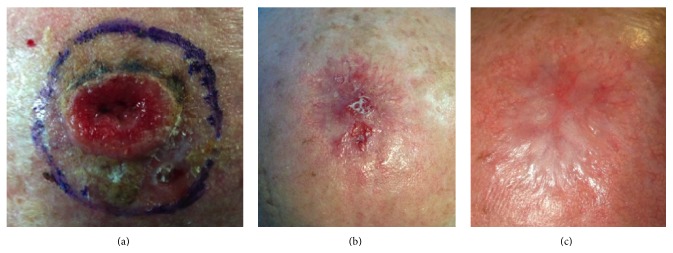
Patient number 1 of group A: (a) preoperative appearance with lesion, (b) postoperative appearance after 25 days, and (c) postoperative appearance after 35 days.

In group A we obtained a score of 8 representing the value of the best clinical scar and a score of 17 for the worst clinical scar. The mean score of the MSS was 13.5 ± 2.6 (SD) representing a very good quality (*p* < 0.05) of the scars in patient with maximum defect size of the lesion of 19.62 cm^2^ (Figure 6).

**Figure 7:**
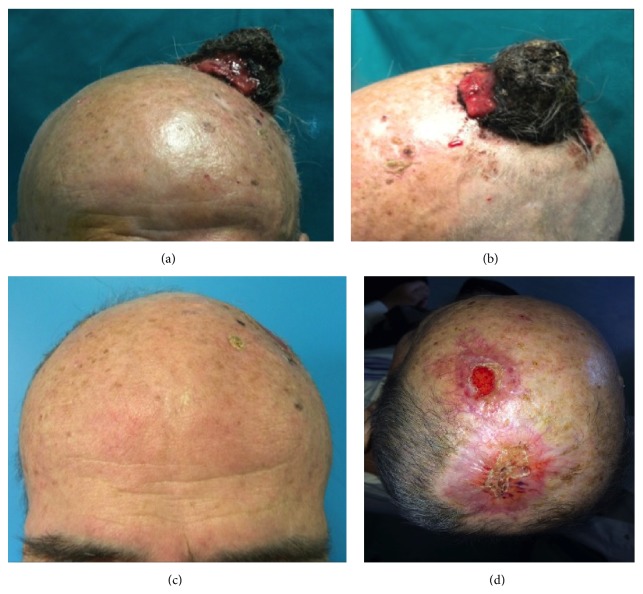
Patient of group B: (a) preoperative appearance with big lesion, (b) detail of the lesion, (c) postoperative appearance in frontal projection after 45 days, and (d) postoperative appearance in back projection after 45 days.

**Figure 8:**
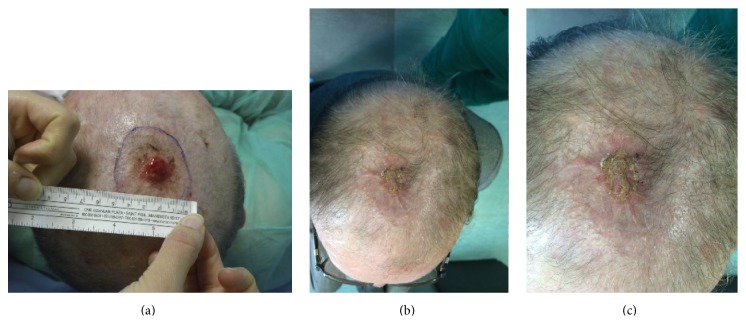
Patient number 2 of group B: (a) preoperative appearance with lesion; 85-year-old patient, affected by chronic lymphocytic leukemia, renal failure, and arterial hypertension; defect size of 65.94 cm^2^; (b) postoperative appearance after reconstruction with pericranial local flap and Integra. After 20 days from surgery the patient had a local recurrence promptly excised and covered with DRT. Initial lesion healed and area of the recurrence not yet healed; (c) detail of lesion.

In group B we obtained a score of 9 representing the value of the best clinical scar and a score of 20 for the worst clinical scar. The mean score of the MSS was 15.1 ± 3.3 (SD) representing a valid result (*p* < 0.05), also in patients with bigger lesions (maximum defect size of 65.94 cm^2^) reconstructed with local pericranial flap and Integra coverage (Figures 7 and 8).

Overall, patients expressed high satisfaction with the results of the DRT reconstruction of the soft-tissue scalp defects.

The average time of the surgery procedure for patients in group A was 30 minutes and in group B was 70 minutes.

In group A neither minor nor major complications were observed.

In group B there was one minor complication and one major complication: one patient under anticoagulant therapy during the 48 hours of postoperative period developed a haematoma, which was promptly drained without further complications, and one patient developed an ulceration of the reconstructed area and had a recurrence after radiotherapy.

The patient in group B with maximum defect size (patient 2) (Figure 8) showed 20 days postoperatively a tumor recurrence near the first lesion that we promptly removed till the galeal fascia and covered with Integra because the dimension of the defect was inferior to 15 cm^2^.

### 3.1. Histological Evaluation

Incisional punch biopsies (3 mm in diameter) at the level of the soft-tissue defects (after the lesion excision) were obtained from a small sample of patients at baseline, week 3, and week 12.

Microscopic evaluation of routinary hematoxylin-eosin stained paraffin-derived sections was performed to morphologically verify the healing process of the loss of substance treated by Integra.

**Figure 9:**
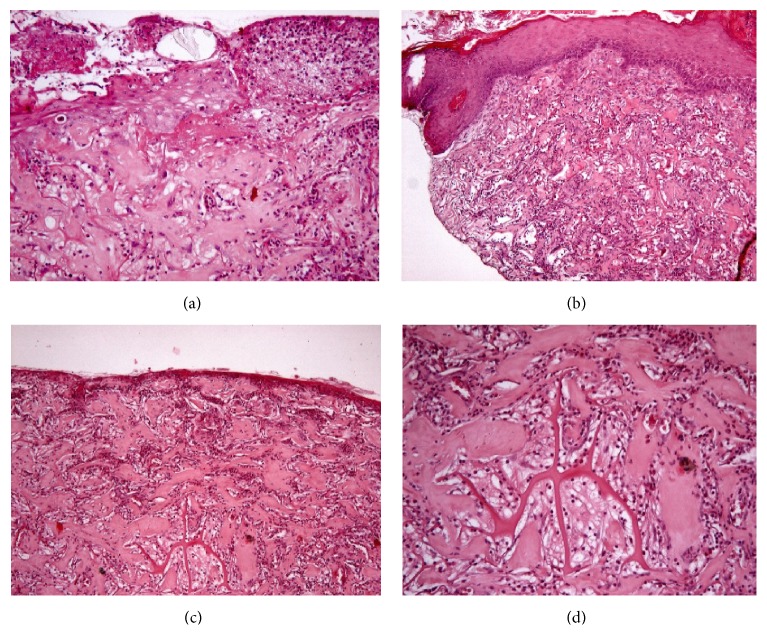
Histological analysis: (a) switching between skin covered by skin and ulcerated area, (b) side with the overlying skin epidermidis, (c) ulcerated area with underlying lattice partially amorphous material, and (d) higher magnification image 1 particularly of amorphous material.

Microscopic evaluation showed a progressive process of wound reepithelialization, starting from an ulcerated area with underlying lattice partially amorphous material (Figures 9(c) and 9(d)), proceeding through the formation of dermal granulation tissue rich in newly deposed vessels with overlying early migration of cheratinocytes with switching area between skin and wound (Figure 9(a)), and finally resulting in overlying skin epidermis.

### 3.2. Statistical Analysis

Values are shown as mean plus standard error of mean (s.e.m.).

Results were analyzed by means of Student's *t*-test, and differences were considered statistically significant for *p* < 0.05.

## 4. Discussion

Reconstruction of postoncologic scalp defects especially in elderly patients with multiple comorbidities presents a challenge for the surgeon.

After excision, reconstruction of full-thickness defect can be performed with differenttechniques including local or locoregional flaps, free flaps, and grafts. New techniques in tissue regeneration are mostly explained in the literature, but there are no articles concerning the possible use of Integra.

The reconstruction of scalp defects is challenging especially in lesions where the bone is exposed. Coverage of the bone with full-thickness skin graft after its burning is a valid technique but not always durable and subsequently radiotherapy can lead to graft ulceration.

Skin graft contraction can lead to adjacent structures distortion with tumor surveillance problems. Without good vascular support, full-thickness skin graft can necrotize and can give coverage only for small areas with an aspect similar to a patch [22].

In our study we decided to use a pericranial locoregional flap to cover the bone in defect >20 cm^2^ because Integra can survive in well-vascularized areas: the periosteum, in fact, is an essential tissue for the healing of wound bed where bone is exposed. The possibility of survival of the graft on the bone without periosteum is almost none [29].

Burning the bone with drill is unreliable and the extension of the neo vascularization is unpredictable. The process of capillaries invasion of the dermal matrix comes from the bed and from the margins of the wound; without periosteum the healing process comes only from the margins of the wound [30].

For all these reasons we chose to cover the exposed bone of the scalp with a full vascularized pericranial flap. Other advantages of bone coverage with pericranial flap and Integra in scalp large defect are the flexibility and pliability of the reconstructed area compared to the fixity of Integra apposed straight to the bone in case of survival of the template [10].

Integra Dermal Regeneration Template consists of a two-layer skin regeneration system. The outer layer is a thin silicone film that acts as the patient's epidermis. The inner layer is made of a complex matrix of cross-linked fibers that acts as a scaffold for regeneration of dermal skin cells. Integra coverage reduces the scar contraction and the development of hypertrophic scar.

We observed in our results that the mean postoperative reepithelization time was 42.5 ± 6.4 (SD) for both group A and group B; this result is essential to understand the basic importance of locoregional pericranial flap to cover the bone avoiding the apposition of dermal substitute straight to the bone. In fact this flap can guarantee the vascularization for the integration of the template and the subsequent growth of new derma.

The Manchester Scar Scales for the two groups showed overlapping results: in group A the mean score was 13.5 ± 2.6 (SD), and in group B the mean score was 15.1 ± 3.3 (SD). Analyzing the results we can assume that reconstruction with Integra double regeneration template in small and large defect sizes can guarantee good scars in terms of color (normal or slight mismatch), skin texture (matte), contour (flush with surrounding skin), distortion (none or mild), and texture (just palpable). The patient's evaluation of the scars in groups A and B was sufficient with a mean result of the VAS of 5.

In our study we chose a healing for second intention for our patient because we did not cover the new derma with skin graft after silicone removal; we preferred this solution to avoid a secondary surgery for the patient and to evaluate the result of healing for second intention.

As shown in other studies Integra sometimes may tend to give hyperpigmented scars; hyperpigmentation may be attributed to an inflammatory reaction combined to erythrocytes extravasation which can be treated with IPL [31].

In our study we treated only elderly patients and the majority of them were bold: this condition has to be evaluated considering that Integra does not support a regrowth of hair in the treated area.

Use of Integra in postoncologic scalp defects offers significant advantages: immediate coverage while waiting for histological exam, thick and durable coverage, morbidity reduction of donor site, immediate accessibility of the product, and good cosmetic result of the reconstructed area.

## 5. Conclusions

In our work we obtained a safe reconstruction, easy to realize, durable, and with favorable and predictable result.

The reconstructed tissue was flexible, long-lasting, uniform in texture, and radiotherapy stable.

Comparing to the reconstruction using only skin graft we observed less contraction or distraction of the tissue and we did not observe hypertrophic scar either.

In conclusion we can assume that the reconstruction with local pericranial flap is a valid technique to guarantee the attachment of the Dermal Regeneration Template in large defect of the scalp with an acceptable functional and aesthetic outcome.

## Figures and Tables

**Table 1:** Group A. Details of 10 patients with mean defect size of 12.51 cm^2^ ± 4.4 (SD).

Group A					
Patients	Age	Gender	Comorbidities/smoke	Defect size (cm^2^)	Tumor type
1	76	M	Arterial hypertension and antiplatelet drugs	4.90	BCC

2	75	M	Coronary heart disease, arterial hypertension, smoking habits, and antiplatelet drugs	7.06	BCC

3	79	M	Diabetes mellitus type II and smoking habits	19.62	BCC

4	72	F	Arterial hypertension	8.24	SCC

5	82	M	Coronary heart disease, arterial hypertension, and antiplatelet drugs	9.42	BCC

6	84	F	Atrial fibrillation, diabetes mellitus type II, arterial hypertension, and permanent anticoagulation	8.83	BCC

7	74	M	Arterial hypertension, renal failure, and smoking habits	12.36	BCC

8	78	M	Arterial hypertension	13.73	BCC

9	80	F	Arterial hypertension, diabetes mellitus type II, and smoking habits	15.7	BCC

10	77	F	Arterial hypertension, coronary heart disease, and antiplatelet drugs	17.66	BCC

**Table 2:** Group B. Details of 10 patients with mean defect size of 28.7 cm^2^  ± 13.5 (SD).

Group B					
Patients	Age	Gender	Comorbidities/smoke	Defect size (cm^2^)	Tumor type
1	83	M	Coronary heart disease, diabetes mellitus type II, and smoking habits	28.26	BCC

2	85	M	Chronic lymphocytic leukemia, renal failure, and arterial hypertension	65.94	SCC

3	79	M	Arterial hypertension and antiplatelet drugs	21.19	BCC

4	73	F	Diabetes mellitus type II and smoking habits	31.4	SCC

5	81	M	Coronary heart disease, arterial hypertension, and antiplatelet drugs	23.55	BCC

6	76	M	Arterial hypertension	21.19	BCC

7	74	M	Diabetes mellitus type II, arterial hypertension, atrial fibrillation, and permanent anticoagulation	22.96	BCC

8	82	F	Coronary heart disease, arterial hypertension, and smoking habits	27.47	SCC

9	89	M	Renal failure and diabetes mellitus type II	21.58	BCC

10	90	M	Renal failure, diabetes mellitus type II, and smoking habits	23.55	BCC

**Table 3:** The results of the Manchester Scar Scales (MSS) and Visual Analogue Scale (VAS) in group A.

Group A								
Patients	Color	Skin texture	Contour	Distortion	Texture	Scar score	VAS score	MSS score
1	1	1	1	1	2	6	8	14
2	1	1	1	1	1	5	9	14
3	3	2	2	2	2	11	6	17
4	2	1	1	1	2	7	7	14
5	1	1	1	1	2	6	2	8
6	1	1	2	1	2	7	3	10
7	2	2	2	2	2	10	5	15
8	2	2	2	2	2	10	5	15
9	3	1	2	2	2	10	5	15
10	1	1	2	2	3	9	4	13
Mean	**1.7**	**1.3**	**1.6**	**1.5**	**2**	**8.1**	**5.4**	13.5
SD							**2.1**	2.6

**Table 4:** The results of the Manchester Scar Scales (MSS) and Visual Analogue Scale (VAS) in group B.

Group B								
Patients	Color	Skin texture	Contour	Distortion	Texture	Scar score	VAS score	MSS score
1	2	1	2	2	2	9	4	13
2	3	2	2	2	3	12	7	19
3	2	2	1	1	2	8	4	12
4	2	2	3	2	2	11	6	17
5	2	1	2	2	2	9	6	15
6	1	2	1	1	1	6	3	9
7	2	2	2	2	2	10	5	15
8	3	1	1	3	3	11	6	17
9	2	1	2	2	2	9	5	14
10	3	2	2	3	3	13	7	20
Mean	**2.2**	**1.6**	**1.8**	**2**	**2.2**	**9.8**	**5.3**	15.1
SD							**1.3**	3.3
